# Predictors of Outcome in Clinically Diagnosed Viral Encephalitis Patients: A 5-Year Prospective Study

**DOI:** 10.1155/2020/2832418

**Published:** 2020-07-08

**Authors:** Guibo Feng, Lunqin Zhou, Feng Li, Yida Hu, Xuefeng Wang, Xin Tian

**Affiliations:** ^1^Department of General Medicine, Yongchuan Hospital of Chongqing Medical University, Chongqing 402160, China; ^2^Department of Neurology, The First Affiliated Hospital of Chongqing Medical University, Chongqing Key Laboratory of Neurology, 1Youyi Road, Chongqing 400016, China

## Abstract

**Background:**

Viral encephalitis is the most common infectious disease of the central nervous system and is associated with high morbidity, mortality, and disability. The objective of this study was to analyze the clinical characteristics, auxiliary examinations, therapeutic management, and outcomes of patients clinically diagnosed with viral encephalitis and identify the outcome predictors.

**Methods:**

We conducted a prospective observational study by collecting information from patients clinically diagnosed with viral encephalitis at the First Affiliated Hospital of Chongqing Medical University and Yongchuan Hospital of Chongqing Medical University from January 2013 to December 2018. Univariate and multivariate analyses were performed to identify factors that influenced good patient outcomes (mRS < 3) and poor patient outcomes (mRS ≥ 3) at discharge.

**Results:**

In total, 216 patients were enrolled in the study. The multivariate analysis suggested that the following factors were associated with a poor outcome: Glasgow Coma Scale (GCS) score (OR 0.154, 95% CI (0.078-0.302), and *P* < 0.001), focal neurological deficits (OR 9.403, 95% CI (1.581-55.928), and *P* = 0.014), and total length of hospital stay (OR 1.119, 95% CI (1.002-1.250), and *P* = 0.045). However, neurological intensive care unit (NICU) treatment, status epilepticus, and abnormal electroencephalogram (EEG) findings did not influence the prognosis of patients.

**Conclusion:**

Our study suggests that low GCS scores at admission, focal neurological deficits at admission, and a prolonged total hospital stay are predictors of a poor outcome at discharge in clinically diagnosed viral encephalitis patients. Whether early and effective neurological rehabilitation can improve the prognosis of viral encephalitis patients with focal neurological deficits remains to be confirmed in further studies.

## 1. Introduction

Viral encephalitis is the most common infectious disease of the central nervous system and occurs worldwide. Viral encephalitis has high morbidity and mortality, seriously threatening the lives and health of the general public [1, 2]. Recently, due to the increased use of antiviral drugs and the utilization of modern intensive care equipment, the mortality rate due to viral encephalitis has decreased to 5-20% [3–6]. In developing countries, approximately 50-60% of surviving patients with an identified cause of viral encephalitis have a poor long-term prognosis [7–9]. Long-term, persistent, neurological, and cognitive sequelae [10] can cause tremendous stress and a substantial financial burden to patients' families and society [4, 11–15]. Therefore, research identifying prognostic factors in viral encephalitis patients can provide a theoretical framework to guide early decision-making and promote timely intervention/treatment and the capability to make better decisions to improve both patient prognosis and quality of life. Such improvements have important clinical and social implications.

The diagnosis of viral encephalitis should consider epidemiological data, clinical manifestations, medical history, and a comprehensive analysis of auxiliary examination results. The gold standard for diagnosis is the detection of viral antigens or specific antibodies in the cerebrospinal fluid (CSF) or the detection of the virus in brain tissue. However, even with modern laboratory testing techniques, the diagnostic rate of viral encephalitis remains low worldwide, and in approximately 70% of cases, the specific causes of viral encephalitis remain unknown [16–18]. In China, the pathogenicity of viral encephalitis is still not widely considered in the diagnosis of viral encephalitis. The diagnosis is based on clinical and related auxiliary examination data. Currently, knowledge regarding the prognosis of viral encephalitis when the microbiological etiology is unknown is limited. In addition, the rate of etiological diagnosis is low, and clinicians have been increasingly interested in identifying factors that can predict an adverse prognosis in clinically diagnosed viral encephalitis patients. In this study, we analyzed features of clinically diagnosed acute viral encephalitis during the acute phase and gathered information regarding examinations, treatment, and other related data to identify factors that have predictive value in patient prognosis. The results of this study can be widely applied to determine the prognosis of patients with cryptogenic viral encephalitis.

In this study, we found that a low Glasgow Coma Scale (GCS) score at admission, focal neurological deficits, and a prolonged total hospital stay were predictors of a poor outcome at discharge. The discovery of the above poor-outcome predictors is important for guiding the early identification of patients who are at the greatest risk of a poor outcome. Timely, targeted treatment of this disease, especially in patients with new-onset focal neurological deficits, is likely to have important ramifications for improving prognosis.

## 2. Methods

### 2.1. Study Design

We prospectively collected the information of patients diagnosed with viral encephalitis at the First Affiliated Hospital of Chongqing Medical University and Yongchuan Hospital of Chongqing Medical University between January 2013 and December 2018. A neurologist examined the patients' medical histories, laboratory results, imaging results, electroencephalogram (EEG) results, and treatment information to further determine the patients' diagnosis. Another neurologist, who was not involved in the collection of the patients' medical histories, performed a prognostic assessment to determine the discharge outcome.

### 2.2. Definitions

According to the International Encephalitis Consortium, encephalitis is diagnostically defined as a persistent altered mental status (altered level of consciousness or personality change) lasting more than 24 hours without encephalopathy from other causes with at least three of the following associated manifestations: fever within 72 hours before and after the onset of symptoms (>38°C), generalized or partial seizures not fully attributable to a preexisting seizure disorder, new onset of focal neurological deficits, increased white blood cell count in the CSF (≥5/mm^3^), and imaging/EEG results consistent with changes associated with encephalitis [2, 19]. Seizures were defined as clinical seizures or confirmed by EEG results. Status epilepticus (SE) was defined as continuous seizure activity lasting longer than 5 min or periods of repeated seizure activity totaling over 5 min without regaining consciousness between seizures [20]. Coma was defined as a GCS score ≤ 8. Each patient's clinical outcome at discharge was graded with a modified Rankin Scale (mRS) score [21]. Patients with mRS scores ≥ 3 were included in the poor-outcome group, whereas patients with mRS scores = 0-2 were included in the good-outcome group [22].

### 2.3. Inclusion/Exclusion Criteria

The inclusion criteria were as follows: age greater than 16 years; acute or subacute onset; and no evidence of bacterial, tubercle bacillus, or fungal infection in the patients meeting the above-listed diagnostic criteria for encephalitis.

The exclusion criteria were as follows: (1) patients with incomplete clinical data; (2) patients diagnosed with other serious diseases, such as malignant tumors, serious infections outside the central nervous system, severely critical organ dysfunction, and cerebral infarction or cerebral hemorrhage; (3) patients with AIDS-induced central nervous system diseases, including toxoplasmosis, cryptococcal meningitis, and HIV-related encephalopathy; (4) patients with encephalopathy secondary to other factors, including septicemia, pyemia, and noninfective factors, such as toxicity or metabolic disease; and (5) patients with encephalitis caused by other causes, such as central nervous system vasculitis, autoimmune encephalitis, and chronic encephalitis.

This study was approved by the ethics committees of the First Affiliated Hospital of Chongqing Medical University and Yongchuan Hospital of Chongqing Medical University; all patients or their family members provided informed consent for participation in the study and signed a consent form allowing their medical archival data to be used for this research.

### 2.4. Data Collection

The following information was collected for all eligible patients: demographic information (sex and age); hospital-related data (total length of stay, whether the patient stayed in the neurological intensive care unit (NICU)); clinical history (time from onset to admission and history of preexisting infections); clinical symptoms (headache, nausea, vomiting, mental and behavioral abnormalities, seizures, and SE); clinical signs (highest body temperature since onset, presence or absence of nuchal rigidity, and presence or absence of new-onset focal neurological deficits); results of initial laboratory tests (routine bloodwork, electrolytes, albumin, and serum creatinine) completed within 24 hours of admission; results of the initial CSF examination (number of cells, number of nucleated cells, protein, and sugar) completed after admission; imaging findings (cerebral computed tomography (CT) or magnetic resonance imaging (MRI)); EEG findings; GCS score within 24 hours of admission; and main treatment data (whether antiviral drugs or glucocorticoids were used), as well as whether mechanical ventilation was required during hospitalization. All patients underwent a complete neurological examination before discharge, and all patients were evaluated to determine their outcome based on the mRS score.

### 2.5. Statistical Methods

The SPSS 21.0 statistical software package was used for the statistical analysis. Normally distributed data are presented as means ± standard deviations, and nonnormally distributed data are reported as medians and interquartile ranges (*M* [P25, P75]). The chi-squared test was performed for the single-factor analysis of count data; the *t*-test was performed for the single-factor analysis of normally distributed data; and the Kruskal-Wallis *H* test was performed for the single-factor analysis of nonnormally distributed data. To identify the statistically significant single-factor indicators, a multivariate logistic regression analysis was performed (*α* = 0.05, *β* = 0.1). The differences were considered statistically significant at *P* < 0.05.

## 3. Results

During the follow-up period, the information of 423 patients with viral encephalitis was collected at the First Affiliated Hospital of Chongqing Medical University and Yongchuan Hospital of Chongqing Medical University. After excluding patients who were younger than 16 years (*n* = 39), patients with incomplete clinical data (*n* = 68), and patients experiencing complications from malignant tumors or encephalitis secondary to other factors (*n* = 100), 216 patients were eventually included in our study.

### 3.1. Characteristics of the Study Participants

The average age of the patients included in the study was 43 (26, 58) years, and 113 (52.31%) patients were men. The average time from onset to admission was 5 (2, 8) days; the average length of stay was 13 (9, 18) days; and 101 (46.76%) patients had a preoperative infection before onset. Most patients presented with at least one common symptom; 146 (67.59%) patients exhibited mental or behavioral abnormalities, 102 (47.22%) patients reported headache, 88 (40.74%) patients had fever, 42 (19.44%) patients had nausea/vomiting, 43 (19.91%) patients experienced seizure, 10 (4.63%) patients had SE, and 23 (10.65%) patients had focal neurological deficits. Among these patients, 73 (33.80%) were admitted to the NICU, and 11 (5.09%) received mechanical ventilation. Infection complications occurred in 42 (19.44%) patients during hospitalization; 210 (97.22%) patients received antiviral therapy, and 140 (64.81%) patients received glucocorticoid therapy. The detailed demographic information, clinical manifestations, and treatment information of the patients included in the follow-up study are shown in Table 1.

### 3.2. Auxiliary Examinations

All patients followed in this study were subjected to CSF and craniocerebral imaging examinations. Twenty-six (12.04%) patients underwent a CT scan. No abnormalities were found. In total, 190 (87.96%) patients underwent MRI scans, and 69 (69/190, 36.32%) scans were abnormal. EEGs were obtained from 187 (86.57%) patients, and 143 (143/187, 76.47%) were abnormal. The imaging, EEG, and CSF examination results are shown in Table 2.

### 3.3. Predicting Prognostic Factors

**Figure 1:**
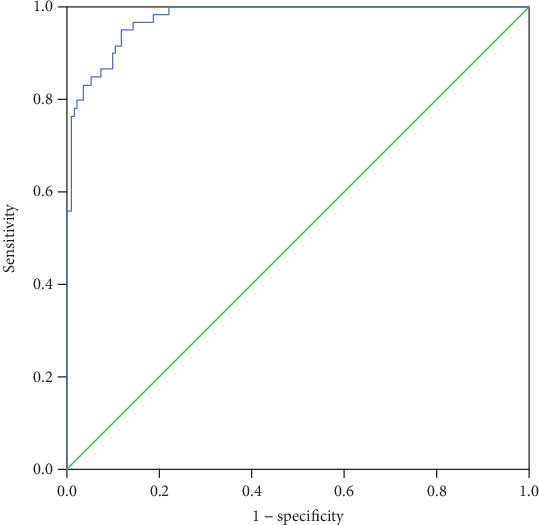
ROC curve of the logistic regression model.

The univariate analysis considered age-related factors (*P* = 0.001), NICU admission (*P* < 0.001), NICU length of stay (*P* < 0.001), total length of hospital stay (*P* < 0.001), mental and behavioral abnormalities (*P* = 0.020), GCS (*P* < 0.001), coma (*P* < 0.001), new-onset focal neurological deficits (*P* < 0.001), SE (*P* < 0.001), seizure (*P* = 0.006), headache (*P* < 0.001), mechanical ventilation (*P* < 0.001), infection complications (*P* < 0.001), glucose in CSF (*P* = 0.013), and radiological findings (*P* < 0.001) (see Tables 1 and 2). The factors associated with a poor outcome in the multivariate analysis were the GCS score (OR 0.154, 95% CI (0.078-0.302), and *P* < 0.001), new-onset focal neurological deficits (OR 9.403, 95% CI (1.581-55.928), and *P* = 0.014), and total hospital stay (OR 1.119, 95% CI (1.002-1.250), and *P* = 0.045) (Table 3). The Hosmer-Lemeshow goodness-of-fit test showed that the model fit well (*χ*^2^ = 4.336, *P* = 0.826). A receiver operating characteristic (ROC) curve was constructed to test the logistic regression equation, and the area under the receiver operating characteristic curve (AUC) was 0.977 (see Table 4 and Figure 1).

## 4. Discussion

In this prospective study, 216 cases of clinically diagnosed viral encephalitis were included. Based on the neurological status assessment at discharge, 59 patients were identified as having a poor outcome at discharge. Through the retrospective analysis of demographic information, clinical features, auxiliary examination results, and treatment information, we found that a low GCS score at hospital admission, new-onset focal neurological deficits, and a prolonged total hospital stay predicted a poor outcome at discharge. However, age, seizure occurrence, SE status, NICU length of stay, mechanical ventilation requirement, cranial imaging findings, and EEG abnormalities did not predict a poor outcome.

The GCS is the most commonly used scale for assessing the functional status of the central nervous system. Lower scores indicate worse brain functioning. In this study, a low GCS score at admission was a predictor of a poor outcome in patients with viral encephalitis, but in the multivariate analysis, a GCS score ≤ 8 was not a predictor of a poor outcome. The effect of the GCS score on the prognosis of viral encephalitis patients has been previously reported. Whitley [23] found that patients with herpes simplex virus encephalitis (HSE) with a GCS score greater than 6 usually had a better prognosis than those with a GCS score less than 6. Kamei et al. [24] also found that a low GCS score before the initiation of acyclovir treatment was an independent predictor of a poor prognosis in HSE patients. Recently, Singh et al. [25] found that coma (GCS score ≤ 8) was associated with poor discharge outcomes in patients with viral encephalitis. In addition, a retrospective study of prognostic factors in 1,107 clinically diagnosed viral encephalitis patients suggested that a low GCS score and coma (GCS score ≤ 8) at admission are predictors of adverse outcomes at discharge, but coma was not a predictor of the long-term prognosis (follow-up at 6 months) [26]. Many studies have confirmed that low GCS scores are highly predictive of a poor prognosis in viral encephalitis patients. Furthermore, calculating the GCS score is simple and fast, can be completed at bedside, and has important significance in guiding the early prediction of a patient's prognosis.

In this prospective study, for the first time, we report that focal neurological deficits can predict a poor outcome in adult patients with viral encephalitis. The occurrence of focal neurological deficits may be due to cerebral edema and blood loss caused by brain damage. Several studies have suggested that Japanese encephalitis (JE) is the most common type of viral encephalitis with a definitive etiology [27]. A link between focal neurological deficits and neurological sequelae has been reported in children with JE [28]. Rayamajhi et al. [29] also reported that focal neurological deficits are independent risk factors for the presence of sequelae upon discharge in hospitalized children with JE, and the sequelae may persist for up to 6 weeks. In this study, 23 (10.65%) patients had focal neurological deficits; this number was lower than that (31.3%) previously reported in children with JE [29]. For patients with focal neurological deficits, whether appropriate early-stage neurological rehabilitation treatment can improve the long-term prognosis will be further discussed in future studies. We found that the age of patients without focal neurological deficits was younger (43 [25, 57]) than that of patients with neurological deficits (52 [33, 62]), but the difference was not significant (*P* = 0.055). Similarly, in the comparison of the imaging results of patients with and without focal neurological deficits, no significant difference was found (*P* = 0.113). This may be related to the small sample size included in this study and the differences in the methods and time points of the relevant imaging examinations in patients with viral encephalitis admitted to two different hospitals in this study. In the future, we will focus on whether imaging conditions (such as the examination method, examination time, lesion site, and lesion side) are related to the prognosis of patients with viral encephalitis with focal neurological deficits.

Patient hospitalization time after admission was determined by the severity of a patient's condition and complications. The hospitalization time of a patient with a poor prognosis is usually longer than that of a patient with a good prognosis [25, 26, 30]. Misra et al. [31] reported that hospitalization time was an independent risk factor for mortality in patients with central nervous system infections in the NICU. Our study also found that a prolonged total hospital stay is a risk factor for a poor prognosis. However, due to the limited number of patients included in this study, we could not further delineate whether prolonged hospital stays were due to the critical conditions of the patients with viral encephalitis or other complications.

Several reports have indicated that older age is a predictor of poor prognosis in patients with HSE [30] and that an age older than 65 years is a predictor of poor prognosis in patients with viral encephalitis [25]. Our study also found that the age of the patients with poor outcomes was significantly higher than that of the patients with good outcomes. However, in this study, the average age of the patients was lower than the average age of the patients included in the abovementioned study. Therefore, the finding that age failed to predict a poor outcome may be related to the relatively young age of the included patients.

Generally, patients with viral encephalitis are admitted to the intensive care unit (ICU) because of respiratory failure, disturbance of consciousness, changes in consciousness due to SE, persistently high fever, or the need for mechanical ventilation. Several reports have suggested that mechanical ventilation [26] and ICU admission [25] are predictors of a poor prognosis in patients with viral encephalitis. This study did not find that mechanical ventilation predicted a poor outcome, which is consistent with the findings reported by Singh et al. [25]. This result may be related to the fact that some patients were unconscious due to SE or complications of severe pulmonary infection and were treated with mechanical ventilation until their complications were effectively controlled without affecting the functional prognosis.

Mental and behavioral abnormalities are common symptoms in patients with viral encephalitis. Some surviving patients may have this sequela for a long period of time. Previous studies have suggested that patients with psychomotor abnormalities usually have a poor prognosis [26]. In this study, 146 (67.59%) patients experienced this symptom; this percentage was higher than the percentages reported in previous studies [13, 26]. Because of the short follow-up period in this prospective study, long-term observations of cognitive and noncognitive function outcomes were not possible. In future studies, we will introduce a more detailed advanced cortical function assessment method to further observe the long-term prognosis of patients with mental and behavioral abnormalities and their ability to return to normal life.

Viral encephalitis is a common cause of epileptic seizures and SE. The incidence of seizures during the acute phase of HSE can be as high as 50%, and the incidence of seizures in JE patients is 7%-46% [32, 33]. Studies have suggested that seizure is a predictor of a poor prognosis [34, 35]. The proportion of patients who experienced seizures in this study was 19.9%, and the incidence of SE was 4.6%, both of which were higher in the poor-outcome group than in the good-outcome group. A previous study suggested that seizures in patients with herpes simplex virus type-1 (HSV-1) encephalitis may be associated with poor clinical outcomes [32]. A retrospective study involving 103 patients with acute encephalitis (28 of whom had viral encephalitis) suggested that encephalitis combined with SE significantly increased the risk of death [36]. However, Singh et al. [37] found that 24.2% of patients with viral encephalitis had seizures during hospitalization, and 34.8% of patients with seizures had a good prognosis; furthermore, postencephalitic epilepsy (PE) did not negatively affect the patients' good prognosis status after 1 year. This finding further confirms that seizures were not a predictor of a poor outcome in our study involving patients with viral encephalitis.

Brain imaging and EEG examinations have certain values in the diagnosis and prognosis of viral encephalitis. Several reports have suggested that the existence of restricted diffusion on MRI and diffusion-weighted imaging is a predictor of a poor prognosis at discharge in patients with HSE [30]. In our study, the abnormal MRI rate in the poor-outcome group (59.3%) was significantly higher than that in the good-outcome group (27.2%), but because our study included different types of viral encephalitis, the imaging examinations may lack specificity. In addition, the time from the onset of symptoms to complete cranial brain imaging was not exactly the same in patients, and no cranial brain imaging abnormalities were found to predict a poor outcome. EEG abnormalities are common in viral encephalitis patients, especially in those with HSE confirmed by PCR. In this study, 187 patients underwent an EEG examination, and the abnormal rate was 76.5%, which was higher than that reported by Zhao et al. [26] However, EEG abnormalities were not valuable in predicting a poor outcome in patients with viral encephalitis.

The early use of antiviral drugs can significantly improve the prognosis of viral encephalitis patients and reduce mortality [7]. Singh et al. [30] reported that the delayed initiation of acyclovir treatment (1 day after admission) was a predictor of a poor prognosis at discharge in patients with HSE. In this study, 210 (97.2%) patients received antiviral treatment, but some patients were referred for admission from other medical institutions; therefore, we did not analyze correlations between types of antiviral drug, initiation times, total treatment times, and prognoses of patients. Currently, the use of glucocorticoids for the treatment of viral encephalitis is controversial, but glucocorticoids can control inflammation and reduce cerebral edema. Some existing animal experiments and clinical observations have found that a combination of glucocorticoids for the treatment of HSE is beneficial for improving prognosis. However, the current evidence does not support this treatment combination as a standardized treatment program [38]. In this study, 140 patients received glucocorticoid therapy, and we did not find a predictive value of glucocorticoid therapy for prognosis. The use of antiviral drug therapy and glucocorticoid therapy in patients with clinically diagnosed viral encephalitis still requires further large-scale, prospective controlled studies to determine their effects on prognosis.

It is worth noting that fever occurred in 41% of patients in this study, which was similar to the fever rate in patients with viral encephalitis without an identified etiology (36%) but lower than the fever rate in patients with an identified infectious etiology (63%) in a recent study [39]. The proportion of patients with fever in our study was higher than that of patients with clinically diagnosed viral encephalitis in another study (20.7%) at the initial stage of onset [26]. The difference in fever rates among patients with viral encephalitis in different studies may be related to differences in etiological characteristics. In addition, this study is aimed at clinically diagnosing viral encephalitis, which may include self-limiting viral infections; these patients may not have obvious fever in the early stage and course of the disease, resulting in the relatively low overall fever rate in our study.

This five-year prospective study has certain limitations. This study was polycentric, and the two hospitals involved in the study were both regional center hospitals. Some patients were referred to these hospitals from other hospitals. Therefore, the characteristics of the cases included in this study, as well as the research findings, are not necessarily universally representative. In addition, the data included in this study were all obtained during the patients' hospital stays. Long-term follow-up of patients' prognoses was not available, and the long-term outcomes of the patients could not be further clarified. In addition, due to the lack of etiological determination in this study, the specific types of viral encephalitis could not be clarified, and the prognoses of different types of viral encephalitis can differ according to type [13]. Therefore, these study results cannot be generalized to specific types of viral encephalitis (e.g., HSE). Despite this, the results provide important reference values for the evaluation of outcomes in patients without an etiological diagnosis and who have an undetermined virus type.

## 5. Conclusion

This study suggests that low GCS scores at admission, focal neurological deficits at admission, and a prolonged total hospital stay are predictors of a poor outcome at discharge in clinically diagnosed viral encephalitis patients.

## Figures and Tables

**Table 1:** Demographics and clinical presentation of viral encephalitis in our cohort.

Variables		All (*n* = 216)	Good outcome (*n* = 157)	Poor outcome (*n* = 59)	*P* value
Sex	Male	113	80	33	0.514
	Female	103	77	26	
Age (years)		43 (26, 58)	42 (25, 55)	52 (35, 66)	0.001
Duration of symptoms before hospitalization (days)		5 (2, 8)	5 (2, 9)	5 (3, 7)	0.873
NICU admission	No	143	115	28	<0.001
	Yes	73	42	31	
Total length of stay in NICU (days)		0 (0, 3)	0 (0, 2)	2 (0, 8)	<0.001
Total length of hospital stay (days)		13 (9, 18)	12 (8, 16)	17 (13, 23)	<0.001
Precursor infection	No	115	81	34	0.428
	Yes	101	76	25	
Mental and behavior disorder	No	70	58	12	0.020
	Yes	146	99	47	
GCS		14 (12, 15)	14 (14, 15)	10 (8, 12)	<0.001
Coma	No	200	155	45	<0.001
	Yes	16	2	14	
Nuchal rigidity	No	159	117	42	0.620
	Yes	57	40	17	
Focal neurological deficits	No	193	150	43	<0.001
	Yes	23	7	16	
Status epilepticus	No	206	155	51	<0.001
	Yes	10	2	8	
Seizures	No	173	133	40	0.006
	Yes	43	24	19	
Headache	No	114	70	44	<0.001
	Yes	102	87	15	
Nausea/vomiting	No	174	123	51	0.180
	Yes	42	34	8	
Fever	No	128	94	34	0.765
	Yes	88	63	25	
Temperature (°C)		37.1 (36.6, 38.5)	37 (36.6, 38.5)	37.2 (36.6, 38.6)	0.395
Mechanical ventilation	No	205	155	50	<0.001
	Yes	11	2	9	
Infection complication	No	174	140	34	<0.001
	Yes	42	17	25	
Steroid treatment	No	76	58	18	0.378
	Yes	140	99	41	
Antiviral treatment	No	6	5	1	0.897
	Yes	210	152	58	

**Table 2:** Initial laboratory results and radiological and EEG findings in a cohort of patients with viral encephalitis.

Variables		All (*n* = 216)	Good outcome (*n* = 157)	Poor outcome (*n* = 59)	*P* value
Blood findings					
Blood potassium (mmol/L)		3.87 ± 0.03	3.87 ± 0.45	3.86 ± 0.49	0.805
Blood sodium (mmol/L)		140 (136, 143)	140 (137, 143)	141 (135, 143)	0.817
Blood chlorine (mmol/L)		103 (100, 105)	103 (100, 105)	103 (98, 105)	0.588
Blood calcium (mmol/L)		2.26 (2.18, 2.35)	2.26 (2.20, 2.35)	2.26 (2.20, 2.34)	0.273
Albumin (g/L)		41 (37, 44)	41 (38, 44)	40 (37, 43)	0.153
Creatinine (*μ*mol/L)		66 (55, 79)	65 (55, 79)	69 (56, 87)	0.157
Platelet (10^9^/L)		215 (172, 259)	212 (176, 263)	219 (158, 255)	0.315
Lymphocyte (10^9^/L)		1.33 (0.99, 1.81)	1.35 (1.04, 1.85)	1.30 (0.91, 1.72)	0.165
White blood cell (10^9^/L)		7.90 (6.31, 10.38)	7.87 (6.09, 9.87)	8.50 (7.03, 11.00)	0.062
Hemoglobin (g/L)		132 (119, 144)	132 (119, 144)	131 (122, 145)	0.843
CSF findings					
CSF cell (10^6^/L)		45 (10, 160)	40 (10, 164)	53 (12, 150)	0.492
CSF leukocytes (10^6^/L)		8 (4, 28)	8 (5, 30)	8 (2, 18)	0.568
CSF protein (g/L)		0.48 (0.34, 0.67)	0.47 (0.32, 0.65)	0.55 (0.38, 0.71)	0.054
CSF glucose (mmol/L)		4 (3.2, 4.4)	4 (3.1, 4.3)	4.1 (3.7, 4.8)	0.013
Blood glucose (mmol/L)		5.9 (5.3, 7)	5.9 (5.4, 7)	5.8 (5.3, 7.3)	0.979
CSF glucose/blood glucose		0.64 (0.57, 0.73)	0.63 (0.56, 0.73)	0.66 (0.62, 0.74)	0.076
CSF pressure		157 (120, 183)	157 (120, 185)	140 (115, 177)	0.096
Radiological findings	CT normal	26	21	5	<0.001
	MRI normal	121	99	22	
	MRI abnormal	69	37	32	
EEG findings	Normal	44	35	9	0.136
	Abnormal	143	97	46	

**Table 3:** Factors associated with a poor outcome in patients diagnosed with viral encephalitis^a^.

Variables	OR	95% CI	*P*
Age	1.035	0.994-1.077	0.094
NICU admission	0.055	0.002-1.563	0.089
Total length of stay in NICU	1.140	0.724-1.795	0.572
Total length of hospital stay	1.119	1.002-1.250	0.045
GCS	0.154	0.078-0.302	<0.001
Coma	0.016	0.000-2.497	0.108
Mental and behavior disorder	0.741	0.121-4.557	0.747
Focal neurological deficits	9.403	1.581-55.928	0.014
Status epilepticus	1.079	0.011-105.734	0.974
Seizures	0.885	0.084-9.353	0.919
Headache	0.983	0.199-4.850	0.984
Mechanical ventilation	0.193	0.001-25.629	0.510
Infection complications	1.048	0.209-5.256	0.955
Radiological findings	1.525	0.499-4.660	0.459
CSF glucose	0.730	0.367-1.450	0.369

OR: odds ratio; CI: confidence interval; NICU: neurological intensive care unit; GCS: Glasgow Coma Scale. ^a^Hosmer-Lemeshow statistics (chi-square = 4.336, *P* = 0.826).

**Table 4:** Receiver operating characteristic curve-related statistical indicators.

AUC	*P* value	95% CI
0.977	<0.001	0.961-0.993

AUC: area under the curve; CI: confidence interval.

## Data Availability

The data used to support the findings of this study are available from the corresponding author upon request.
